# *Pten* is a key intrinsic factor regulating raphe 5-HT neuronal plasticity and depressive behaviors in mice

**DOI:** 10.1038/s41398-021-01303-z

**Published:** 2021-03-26

**Authors:** Ling Chen, Wan-Kun Gong, Cui-ping Yang, Chan-Chan Shao, Ning-Ning Song, Jia-Yin Chen, Li-Qiang Zhou, Kun-Shan Zhang, Siguang Li, Zhili Huang, Gal Richter-Levin, Lin Xu, Yu-Qiang Ding

**Affiliations:** 1 Shanghai Pudong New Area Mental Health Center, Tongji University School of Medicine, 200124 Shanghai, China; 2 Key Laboratory of Arrhythmias, Ministry of Education of China, East Hospital, and Department of Anatomy and Neurobiology, Tongji University School of Medicine, 200092 Shanghai, China; 3 State Key Laboratory of Medical Neurobiology and MOE Frontiers Center for Brain Science, Institute of Brain Science, Fudan University, 200032 Shanghai, China; 4 Stem Cell Translational Research Center, Tongji Hospital, Tongji University School of Medicine, 200065 Shanghai, China; 5 Department of Neurobiology and Ethology, and Department of Psychology, University of Haifa, 3498838 Haifa, Israel; 6 Key Laboratory of Animal Models and Human Disease Mechanisms, and Laboratory of Learning and Memory, Kunming Institute of Zoology, The Chinese Academy of Sciences, 650223 Kunming, China; 7 Department of Laboratory Animal Science, Fudan University, 200032 Shanghai, China

**Keywords:** Psychiatric disorders, Molecular neuroscience

## Abstract

Serotonin (5-HT)-based antidepressants, selective serotonin reuptake inhibitors (SSRIs) aim to enhance serotonergic activity by blocking its reuptake. We propose PTEN as a target for an alternative approach for regulating 5-HT neuron activity in the brain and depressive behaviors. We show that PTEN is elevated in central 5-HT neurons in the raphe nucleus by chronic stress in mice, and selective deletion of *Pten* in the 5-HT neurons induces its structural plasticity shown by increases of dendritic branching and density of PSD95-positive puncta in the dendrites. 5-HT levels are elevated and electrical stimulation of raphe neurons evokes more 5-HT release in the brain of condition knockout (cKO) mice with *Pten-*deficient 5-HT neurons. In addition, the 5-HT neurons remain normal electrophysiological properties but have increased excitatory synaptic inputs. Single-cell RNA sequencing revealed gene transcript alterations that may underlay morphological and functional changes in *Pten*-deficient 5-HT neurons. Finally, *Pten* cKO mice and wild-type mice treated with systemic application of PTEN inhibitor display reduced depression-like behaviors. Thus, PTEN is an intrinsic regulator of 5-HT neuron activity, representing a novel therapeutic strategy for producing antidepressant action.

## Introduction

Depression is a common psychiatric illness-causing enormous personal suffering and societal economic burden^1,2^. The currently available antidepressants, such as selective serotonin (5-HT) reuptake inhibitors (SSRIs), were developed based on the “monoamine hypothesis”, which was based on the suggested mechanism of action of earlier drugs (tricyclic antidepressants) and posited that decreased monoamine functions in the brain lead to depression^1,3^. Although it is not clear how decreased monoamine functions contribute to the development of depression, the current antidepressants may exert their therapeutic effects via producing secondary neuroplasticity, as well as upregulating brain-derived neurotrophic factor (BDNF) and adult hippocampal neurogenesis by increasing the number of monoamines at synaptic site^1,4–6^. Monoamine-based antidepressants are known to have a therapeutic delay and unsatisfying remission rate, but they remain the first-line therapy of depression, mainly due to a lack of more effective alternatives. We set out to explore a new way of increasing central 5-HT neuronal plasticity that may produce antidepressant action.

PTEN is a well-known tumor suppressor that acts as a phosphatase for the lipid signaling intermediate phosphatidylinositol-3,4,5-triphosphate, and that inactivates the phosphatidyl inositide 3-kinase (PI3K)/protein kinase B (Akt)/mammalian target of rapamycin complex 1 (mTORC1) signaling pathway^7,8^. In the central nervous system, PTEN is implicated in the regulation of neuronal process complexity and soma size^9,10^ and synaptic plasticity^11,12^. It has been reported that PTEN levels are increased while PI3K and Akt activities are reduced in the cerebral cortex and hippocampus in depressed suicide victims^13,14^. In addition, chronic stress, a known risk factor associated with the development of depression, causes neuronal atrophy including the reduction of synapse number and synaptic functions^15^. We thus hypothesized that chronic stress may increase PTEN activity in depression-associated brain regions, which leads to maladaptive neuroplasticity and depression. Conversely, reducing PTEN activity may reverse these processes and induce antidepressant effects. Towards that end, we focused on central 5-HT neurons and investigated whether PTEN regulates their morphological and functional plasticity, as well as depression-like behaviors in mice.

## Materials and methods

### Animals

Mice were housed in a temperature and humidity-controlled environment with a 12:12-h light/dark cycle (light on at 07:00 a.m.) and were given access to food and water ad libitum. For conditional deletion of *Pten* in central 5-HT neurons, *Pten*^*flox/flox*^ mice (006440, Jackson Laboratories, USA) were crossed with *Pet1-Cre* mice^16^ and *Pet1-Cre; Pten*^*flox/flox*^ mice (referred to as PTEN cKO) were obtained. Littermates with other genotypes (i.e., *Pet1-Cre, Pten*^*+/flox*^, and *Pten*^*flox/flox*^) were used as controls. Animal care practices and all experiments were reviewed and approved by the Laboratory Animal Committee of Tongji University School of Medicine, Shanghai, China (TJmed-010-10). We made full efforts to minimize the use of animals and to optimize their comfort.

### Chronic stress model

Mice (C57B6, male, 6 weeks old) were restrained for 3–4 h daily, followed by 5-min home-cage horizontal shaking 3–5 times in 30–90-min intervals, and control mice were raised in the home cage as usual. The body weight was measured every week and forced swimming tests and sucrose preference tests were conducted at the end of the second week. Four weeks after the beginning of daily stressful treatment, mice were subjected to the examination of depression-like and anxiety-like behaviors.

### Immunohistochemistry

Immunohistochemical staining was performed following the procedure described previously^17^. Briefly, brains were fixed in 4% paraformaldehyde (PFA) in 0.1 M phosphate-buffered saline (PBS; pH7.4) for 24 h, cryoprotected with 30% sucrose in PBS, and cut into 30 μm-thick transverse brain sections that were incubated with primary antibody at 4 °C overnight. After washing in PBS, sections were incubated with secondary antibody for 3 h at room temperature and finally with Cy3-conjugated streptavidin (1:1000, Jackson ImmunoResearch, USA). The following primary antibodies were used: rabbit anti-tryptophan hydroxylase 2 (TPH2; 1:1000, Thermo Scientific, USA), rabbit anti-PTEN (1:1000, Abcam, USA), goat anti-5-HT (1:1000, ImmunoStar, USA), guinea pig anti-5-HT transporter (SERT; 1:1000, FRONTIER INSTITUTE, Japan) and rabbit ant-pAkt (1:1000, Cell Signaling, USA).

Ten mice (5 control and 5 *Pten* cKO) were used to quantify TPH2^+^ morphological characteristics of TPH2^+^ neurons and SERT^+^ axons/terminals in the hippocampus and prefrontal cortex. TPH2^+^ areas in the dorsal raphe nucleus (DRN) and diameters of 20 randomly-selected TPH2^+^ neurons were measured in every sixth section that covered the whole rostro-caudal extent of the DRN using ImageJ software. The optical density of SERT immunoreactivity visualized with diaminobenzidine as chromogen was measured in the hippocampus and prefrontal cortex, and the length of SERT^+^ axons/terminals shown by SERT immunofluorescence in these two regions were measured in the same way (every sixth section).

### Electrophysiology

For clear identification of 5-HT neurons, *Pet1-Cre* mice and *Pet1-Cre;Pten*^*flox/flox*^ mice were crossed with *Rosa-stop-YFP* mice^18^ to get *Pet1-Cre;Rosa-YFP* mice (control) and *Pet1-Cre;Pten*^*flox/flox*^*;Rosa-stop-YFP* mice, which have YFP expression in 5-HT neurons. Coronal slices including the DRN (250 μm thick) were prepared according to the procedures described previously^19^. Under anesthesia with chloral hydrate, the brain stem was cooled and sliced in an ice-cold solution containing (in mM): NaCl, 87; sucrose, 75; KCl, 2.5; CaCl_2_, 0.5; MgCl_2_, 7; NaH_2_PO_4_, 1.25; NaHCO_3_, 25 and glucose, 20; bubbled with 95% O_2_ and 5% CO_2_ using a vibratome (VT1200S, Leica, Germany). Slices were allowed to recover for at least 1 h at 35 ± 1 °C in recording artificial cerebrospinal fluid (ACSF) containing (in mM): NaCl, 124; KCl, 3; CaCl_2_, 2.5; MgSO_4_, 1.2; NaH_2_PO_4_, 1.23; NaHCO_3_, 26, and glucose, 10; bubbled with 95% O_2_ and 5% CO_2_. YFP^+^ cells were visually identified under an upright microscope (Nikon), and whole-cell recording was made from the cells in the dorsomedial and ventromedial DRN^20,21^. Patch pipettes (3–6 MΩ) were filled with an internal solution with the following composition (in mM): K-gluconate, 135; CaCl_2_, 0.5; MgCl_2_, 2; KCl, 5; EGTA, 5; HEPES, 5 (pH 7.3, adjusted with KOH). Membrane currents or voltages were recorded with Multiclamp 700B amplifier, Digidata 1440A converter (Molecular Devices, USA). The signals were digitized at 10 kHz. Clampex 10.4 software (Molecular Devices, USA) was used for control of voltage and data acquisition. All chemicals were purchased from Sigma-Aldrich (USA). Off-line data analysis was performed using Clampfit 10.4 (Molecular Devices, USA) and OriginPro 7.0 (OriginLab Corporation, USA). Resting membrane potentials (RMP) were measured under a current clamp with current holding to 0 pA. Action potential (AP) characteristics were determined from action potentials generated by injecting just enough current to elicit a single action potential^21^. AP amplitude and AHP amplitude were measured in relation to the AP threshold. Frequency–intensity plots were obtained by measuring the number of action potentials generated by depolarizing current steps ranging from 20 pA to 160 pA in 20 pA increments.

For analysis of the dendrite tree of 5-HT neurons, biocytin (0.1%, Sigma) was added to the internal solution in some recordings. After fixation with paraformaldehyde, the slice was sectioned into 30-thick sections, and processed for immunostaining of PSD-95 (1:1000, ThermoFisher, USA) as described above; biocytin was visualized with Cy3-conjugated avidin (1:1000, Jackson ImmunoResearch, USA). Diameters of primary dendrites of Cy3-labeled 5-HT neurons and density of PSD-95^+^ puncta on them were quantified (9 neurons in each group).

### Single-cell RNA sequencing and data analysis

According to the procedure described previously^22,23^, patch electrodes with tips of 5–10 μm diameters were pulled and advanced towards the YFP-positive 5-HT neurons in the acute brain slice. Positive pressure was applied until the tips were next to the cells. Slight negative pressure was then applied until the entire somatic compartment was aspirated into the recording pipette. Immediately, the samples (less than 0.5 μl) were ejected into lysis buffer (4.5 μl), and reverse transcription was performed using a SMARTer Ultra Low RNA Kit (Clontech, Japan). Single-cell cDNA was amplified using an Advantage 2 PCR Kit (Clontech, Japan) according to the manufacturer’s protocol. Purified cDNA from individual cells was sequenced using the Illumina HiSeq 2000 platform. Sequence reads were aligned to the mouse mm9 genome using TopHat. Differential expression analysis was performed using DESeq (Bioconductor) followed by Student *t*-test to model the experimental and gene-specific dispersion, respectively. Genes were considered differentially expressed if *p* < 0.05. Ontological and network analyses of differentially expressed genes were performed using Ingenuity Pathway Analysis software.

### High-performance liquid chromatography (HPLC)

Adult (5 months) wild-type and *Pten* cKO mice were used for HPLC as described previously. In brief, animals were anesthetized with an overdose of chloral hydrate (350 mg/kg body weight), and brains were removed. Tissues of the prefrontal cortex, hippocampus, and midbrain containing the DRN were collected. 5-HT and its metabolite 5-hydroxyindoleacetic acid (5-HIAA) were measured using HPLC electrochemical detection.

### Amperometric detection of 5-HT signals with carbon fiber electrode

5-HT signal was detected with a carbon fiber electrode according to the procedure described previously^24^. A carbon-fiber electrode (CFE) was used as the amperometry working electrode. Each CFE was manually made by inserting a 7 μm carbon fiber into a 1.5 mm × 10 cm glass capillary. The glass capillary was then pulled with a vertical puller. Each CEF had an overall length of 45 mm, with a 100-μm sensor tip of naked carbon fiber. The mice were anesthetized with sodium pentobarbital (50 mg/kg) intraperitoneally (i.p.) and then mounted on a stereotaxic apparatus for surgery. A bipolar stainless steel electrode with a diameter of 1.0 mm sent electrical stimulation into dorsal raphe (AP − 4.3 mm, ML ± 0.2 mm, DV −3.0 mm). A CFE was inserted into the hippocampus (AP −2.1 mm, ML ± 1.7 mm, DV −1.5 mm) for detecting the extracellular 5-HT signal. A patch-clamp amplifier was used under the voltage-clamp mode, with the gain of 0.5 mV/pA and a CEF voltage of a constant +700 mV for amperometry. 5-HT release signal was analyzed by measuring the maximum amplitude of the current recorded with CEF after electrical stimulation (1.0 mA, 20 Hz, 10 pules) in the dorsal raphe nucleus. For each mouse, three successive stimulations with an interval of 5 min were given in dorsal raphe and the maximum amplitudes of the current recorded in the hippocampus were measured, respectively.

### Western blotting

The Midbrain tissue region containing the DRN was prepared as mentioned above. The total proteins were extracted using RIPA buffer (Thermo Scientific, USA) The proteins were separated by standard sodium dodecyl sulfate-polyacrylamide gel electrophoresis (SDS-PAGE) and transferred onto nitrocellulose blotting membranes (GE Healthcare, Germany). The nitrocellulose membranes were incubated with primary antibodies, including polyclonal rabbit anti-phospho-Akt (Ser473) antibody (1:1000, Cell Signaling, USA), rabbit anti-Akt antibody (1:2000, Cell Signaling, USA), rabbit anti-phospho-mTOR (Ser2448) antibody (1:1000, Cell Signaling, USA), rabbit anti-mTOR antibody (1:1000, Abcam, USA), mouse anti-PSD95 (Invitrogen, USA), rabbit anti-Synapsin I (Abcam, USA), rabbit anti-phospho-4E-BP1 (Cell Signaling, USA), mouse anti-Arc antibody (Santa Cruz Biotechnology, USA) and mouse anti-GAPDH antibody (Santa Cruz Biotechnology, USA) overnight at 4 °C, and then with horseradish peroxidase (HRP)-conjugated secondary antibodies (1:1000, KangChen, China) for 1 h at room temperature. The labeled proteins were visualized using the Enhanced Chemiluminescence Kit (Millipore, USA).

### Behavioral studies

#### Open field test

The open field is a square chamber (L × W × H: 28 × 28 × 20 cm) equipped with infrared beams and enclosed in a ventilated and sound-attenuated box illuminated at 50 lux (Med Associates, USA). Animals were gently placed individually in the center of the open field arena and allowed to move freely for 30 min^25^, and the average velocity, the total ambulatory distance in the last 15 min were scored. In all behavior tests, male mice were used.

#### Dark-light choice test

The light-dark exploration test was conducted as described previously^26^. The apparatus consisted of two compartments (dark box, 30 × 20 × 30 cm; lightbox, 30 × 30 × 30 cm) connected by a 6 cm wide by 6 cm high opening located centrally at floor level. Mice were individually placed in the black compartment, and allowed to explore the apparatus for 5 min. The latency of the first entry into lightbox and time spent in the lightbox was evaluated.

#### Elevated plus maze test

The elevated plus-maze is 40 cm above the floor and consists of two open arms (30 × 6 cm) and two enclosed arms (30 × 6 × 15 cm) with open roofs. Each animal was placed in the central platform, facing a closed arm, and tested for 5 min. An entry was counted when four paws of the mouse entered an open or closed arm. The latency to enter open arms and the total time spent in open arms were measured in each test.

#### Novelty-suppressed feeding test

The novelty-suppressed feeding test consists of a 42 × 42 cm open arena with a food pellet placed in the center. Food-deprived mice (24 h) are placed in the arena and the latency to begin chewing food is recorded. Chewing is scored when the mouse is sitting on its haunches and biting with the use of forepaws (sniffing or simply touching the food was not scored), as described previously^27^. Immediately after beginning to eat the chow, the tested animal was placed alone in its home cage with a piece of chow and the amount of food consumed in 5 min is determined by weighing the piece of chow.

#### Contextual fear conditioning

Contextual fear conditioning was conducted according to procedures described previously^16^. Mice were placed in the box and allowed to freely explore for 2 min before receiving five-foot shocks (0.8 mA, 2 s) with intershock intervals of 2 min (Coulbourn Instrument, USA). Mice were then placed back in their home cages 2 min after the final foot shock. Freezing behavior was measured as the amount of time exhibiting freezing behavior during each intershock interval. To study contextual fear memory, mice were placed in the conditioned fear context for 30 min, 1 day, and 8 days after fear conditioning, and their contextual freezing behavior was measured for 10 min without any foot shocks.

#### Tail suspension test

In brief, each mouse was suspended on the edge of a rod 50 cm above a table-top using adhesive Scotch tape, placed approximately 1 cm from the tip of the tail. Tail climbing was prevented by passing the mouse’s tail through a small plastic cylinder prior to suspension. The duration of immobility was manually measured for the last 4 min in the 6 min test period. Mice were considered immobile only when they hung down passively and were completely motionless^28^.

#### Forced swimming test

Mice were forced to swim for 6 min in a cylindrical glass tank (25 cm in height, 10 cm in diameter) that was filled with water to a depth of 19 cm and maintained at 25 ± 1 °C. The time of remaining immobile within the last 4 min of the test was recorded. Mice were judged as immobile when they stopped struggling and floated motionless in the water, making only the movements for keeping their head above water. The mice were then removed from the water, dried with a towel, and returned immediately to their home cage. The FST was used to assess behavioral immobility of mice as a selective standard animal test for antidepressant treatment^29^.

#### Sucrose preference test

Sucrose preference test was performed as described previously with minor modification^30,31^. Mice were acclimated to two bottles containing tap water for 3 days and then were deprived of water for 12 h. Immediately following the deprivation, the mice were given free access to the 2 bottles: one containing tap water and the other containing a 1% sucrose solution for 4 h. The amount of water and 1% sucrose consumed during the 4 h was measured and sucrose preference was calculated as (sucrose consumed − water consumed)/total liquid consumed.

### AAV virus injection

Cre-dependent adeno-associated virus (AAV) expressing cytoplasmic tdTomato and presynaptic synaptophysin-fused EGFP (0.8 μl, Shanghai Taitool Bioscience, China) was injected into the DRN of *Pet1*-Cre and *Pten* cKO mice. Mice were sacrificed 30 days after the injection. The ensity of synaptophysin-fused EGFP-labeled puncta within tdTomato-labeled dendrites in the ventromedial prefrontal cortex of control and *Pten* cKO mice were quantified.

### Drug administration

PTEN inhibitor bpV (pic) (Sigma-Aldrich, USA) was freshly dissolved in sterilized saline and injected through intraperitoneal route (2 mg/kg body weight) once per day for 3 days or 4 weeks.

### Statistics

Contextual fear conditioning and retrieval data were analyzed with two-way ANOVA followed by Sidak’s multiple comparisons test. Immunochemistry, western blotting, electrophysiology, RNA-seq data, and other behavioral data were analyzed by Student’s *t*-test. Data passed the normality test and two-tailed analyses were carried out with a significance level established at *p* < 0.05. Animals of each genotype were randomly assigned to different studies. The graphs represent mean values with error bars representing SEM. The statistical analysis was performed using GraphPad Prism 8.0 (GraphPad Software).

## Results

### Chronic stress increases PTEN expression in 5-HT neurons

**Fig. 1:**
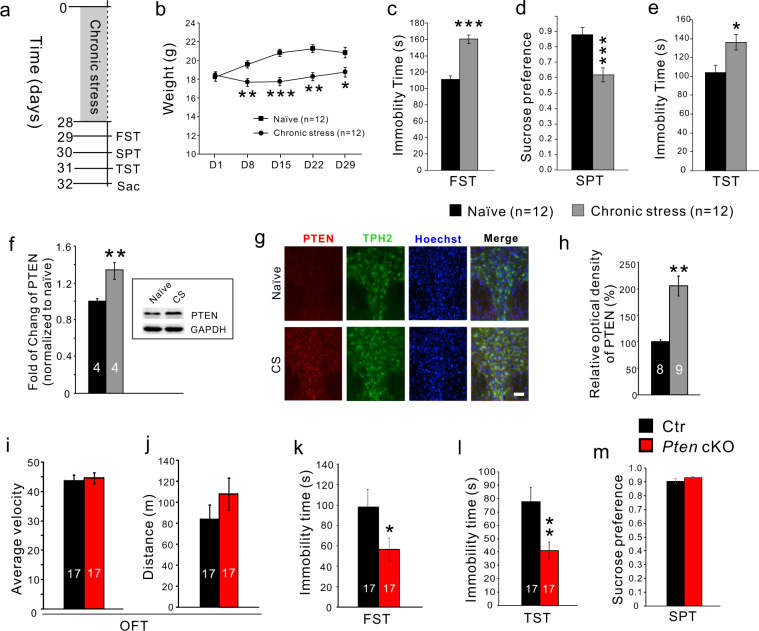
Chronic stress increased PTEN expression in 5-HT neurons and specific knockout of *Pten* in 5-HT neurons induced lower depression-like behavior in mice. **a** Experimental design of chronic stress. Mice were sacrificed (Sac) 1 day after behavioral tests. FST, force swimming test; SPT, sucrose preference test, TST tail suspension test. **b** Increase in body weight is prevented during 4-week chronic restraint. **c**. Immobility time in FST is increased in mice with the 4-week treatment relative to controls. **d** SPT shows a reduction of the preference in mice with the 4-week treatment. **e** Immobility time in TST is increased in mice with the 4-week treatment relative to controls. **f** Western blot shows the elevation of PTEN in midbrain tissues of chronic stress-treated mice. **g** Immunostaining shows the elevation of PTEN in 5-HT neurons in the dorsal raphe nucleus of mice treated with chronic stress (CS). Scale bar = 100 μm. **h** Comparison of PTEN immunofluorescence density in 5-HT neurons. **i**, **j** Average velocity and ambulatory distance in open field tests are comparable between control and *Pten* cKO mice. OFT, open field test. **k, l** Immobility time is reduced in the force swimming test and tail suspension test in *Pten* cKO mice compared with controls. **m** SPT shows no difference between control and *Pten* cKO mice.

Chronic stress is widely used to study depression-like behaviors in rodents^32^. Mice were restrained daily for 3–4 h followed by 5-min-horizontal shaking for 4–5 times over 4 weeks (Fig. 1a). The chronic stress treatment prevented an increase in body weight during the 4-week period relative to controls (Fig. 1b). Depression-like behavior was measured by reduced sucrose preference (Fig. 1d), increased duration of immobility in the forced swim test (Fig. 1c), and tail suspension test (Fig. 1e). One day after the last behavior test, the mice were sacrificed for the Western blot of PTEN. We detected an increase of PTEN in midbrain tissue containing the DRN of chronic stress-treated mice (Fig. 1f). To confirm the elevation of PTEN occurred in 5-HT neurons, we performed double immunostaining of PTEN and TPH2 and found that the density of PTEN immunofluorescence was increased in 5-HT neurons of chronic stress-treated mice (Fig. 1g, h). Thus, chronic stress increases PTEN levels in the DRN 5-HT neurons.

### Reduced depression-like behaviors in *Pten* cKO mice

To investigate the roles of PTEN in central 5-HT neurons, we generated *Pet1-Cre;Pten*^*flox/flox*^ (*Pten* cKO) mice, in which *Pten* is selectively deleted in 5-HT neurons in the brain (Fig. S1). Adult *Pten* cKO mice displayed normal body weight and their brains showed no gross morphological abnormalities (Fig. S2). To investigate possible functional consequences, we performed the following behavioral tests in male *Pten* cKO mice. The open-field test was used to examine spontaneous locomotor activity and there were no obvious alterations in *Pten* cKO mice as shown by similar averaged velocity (Fig. 1i) and total ambulatory distance compared to control mice (Fig. 1j). The forced swimming and tail suspension tests were used to examine depression-like behavioral despair. *Pten* cKO mice displayed less immobility time in the two tests compared with controls (Fig. 1k, l). Anhedonia measured by sucrose preference test showed no difference between control and *Pten* cKO mice (Fig. 1m). Thus, depression-like behaviors are reduced in *Pten* cKO mice, suggesting PTEN in 5-HT neurons is important in regulating depression-like behavior. In addition, anxiety-like behavior measured by the elevated plus-maze (Fig. S3a, b) and novelty suppressed feeding tests (Fig. S3c, d) displayed a reduction in *Pten* cKO mice compared with control mice.

5-HT system plays important role in modulating Pavlovian fear conditioning^33^, and central 5-HT deficiency leads to the fast acquisition and enhanced expression of contextual fear memory in mice^16^. Freezing behaviors in this test can be explained by stressful stimuli-induced behavioral responses. During fear conditioning, *Pten* cKO mice displayed lowered freezing levels after 2nd–5th foot shock relative to controls (Fig. S3f). When placed back into the conditioned context without foot shock, *Pten* cKO mice froze for a much shorter duration than control mice at 30 min, 1 day, and 8 days after conditioning (Fig. S3g). A separate group of mice received foot shocks starting with 0.06 mA and increased in 0.05 mA increments until thresholds of two responses (vocalize and jump) were reached^34^. There were no significant differences in the thresholds between control and cKO mice (Fig. S3h), showing unchanged sensitivity to foot shock in *Pten* cKO mice. In addition, *Pten* cKO mice showed similar withdrawal latency in the tail immersion experiment compared with controls (Fig. S3e), suggesting that thermal sensation is not obviously affected in *Pten* cKO mice. The lowered anxiety level could account for the reduction of acquisition and retrieval of contextual fear memory, but it can also be explained by high resilience to stressful events in *Pten* cKO mice.

### Increased soma size and upregulation of the genes regulating cell size and axonal extension in *Pten* deficient 5-HT neurons

**Fig. 2:**
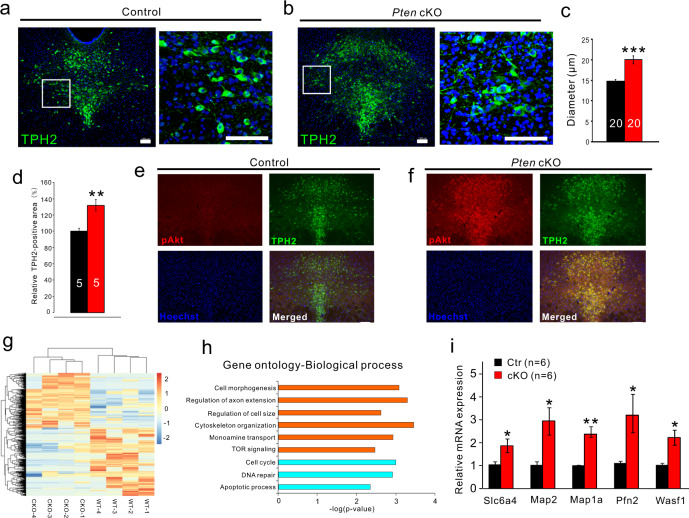
Increased soma size and upregulation of the genes involved in regulating cell size and axonal extension in *Pten*-deficient 5-HT neurons. **a, b** TPH2 immunostaining shows the distribution of 5-HT neurons in the dorsal raphe nucleus of control and *Pten* cKO mice. Scale bars = 100 μm. **c** Soma diameter of TPH2^+^ neurons (*N* = 4 animals, *n* = 20 neurons for statistics analysis) is increased in *Pten* cKO mice compared with controls. **d** Areas containing TPH2 fluorescence are increased in *Pten* cKO mice compared with controls. **e, f** Double immunostaining of TPH2 and pAkt shows a dramatic elevation of pAkt in 5-HT neurons in the dorsal raphe nucleus of *Pten* cKO mice compared with controls. Scale bars = 100 μm. **g** Heatmap depicting differentially expressed genes (DEGs) determined by genome-wide RNA sequencing (RNA-Seq) of control and *Pten*-deficient 5-HT neurons. Rows represent DEGs and columns represent transcriptomic profiles of individual 5-HT neurons. **h** Ingenuity Gene Ontology Analysis depicting to which biological processes the DEGs contribute (orange, upregulated in *Pten*-deficient 5-HT neurons; light blue, downregulated in *Pten*-deficient 5-HT neurons). **i** Upregulation of *Slc6a4*, *Map2*, *Map1a*, *Pfn2*, and *Wasf1* in *Pten*-deficient 5-HT neurons is confirmed by single-cell RT-PCR. All the data are presented as the mean ± s.e.m. *p < 0.05, **p < 0.01, ***p < 0.001. The numbers of animals used are indicated except (**e**, **f**).

Immunostaining of TPH2 showed that the soma size of 5-HT neurons in the DRN and DRN territory containing 5-HT neurons were dramatically increased in *Pten* cKO mice relative to controls (Fig. 2a–d). Given PTEN is a negative regulator of Akt/mTOR signaling pathway, it is expected that elevates pAkt immunoreactivity was present in 5-HT neurons of *Pten* cKO mice (Fig. 2e, f).

To gain molecular insight, we analyzed differences in the gene expression profile between *Pten*-deficient and control 5-HT neurons using the single-cell RNA sequencing technique. The somatic compartments of individual YFP-labeled 5-HT neurons in acute brain slices were harvested for RNA sequencing. The RNA populations from these cells were processed through 3 rounds of amplification. Four cells from wild-type mice and 4 cells from *Pten* cKO mice were selected and subjected to Illumina RNA sequencing. Each of the cells had ≥2 × 10^7^ reads mapping back to the mouse genome when analyzed using the RUM mapping program. We found 859 genes (381 upregulated, 478 downregulated) were differentially expressed in 5-HT neurons of *Pten* cKO mice (Fig. 2g). Gene ontology of differentially expressed genes revealed the biological processes upregulated and downregulated in *Pten*-deficient 5-HT neurons. The absence of *Pten* in 5-HT neurons was associated with significantly increased expression of genes involved in the regulation of axonal extension and cell size, cell morphogenesis, and cytoskeleton organization (Fig. 2h), consistent with the observed phenotype of the enlarged cell body. Some of them such as increases of *Map2*, *Map1a*, *Pfn2*, and *Wasf1* transcripts were confirmed by single-cell RT-PCR, relative to *Gapdh* (Fig. 2i). On the other hand, there was a decrease in the expression of genes involved in the cell cycle, DNA repair, and apoptotic process (Fig. 2h).

Among genes uniquely associated with 5-HT neurons, *Slc6a4* (*Sert*) was increased (Fig. 2i), while *Aadc*, *Vmat2*, and *Htr1a* remained unchanged, suggesting *Pten*-deficient neurons keep the key genes for maintaining its functions, and upregulation of *Sert* may be a consequence of the increased release of 5-HT. Their cellular distribution in the DRN was revealed by in situ hybridization (Fig. S4). Taken together, the alteration of the gene expression profile contributes to the morphological changes of *Pten-*deficient 5-HT neurons.

### Dendritic complexity and excitatory input are increased in *Pten*-deficient 5-HT neurons

**Fig. 3:**
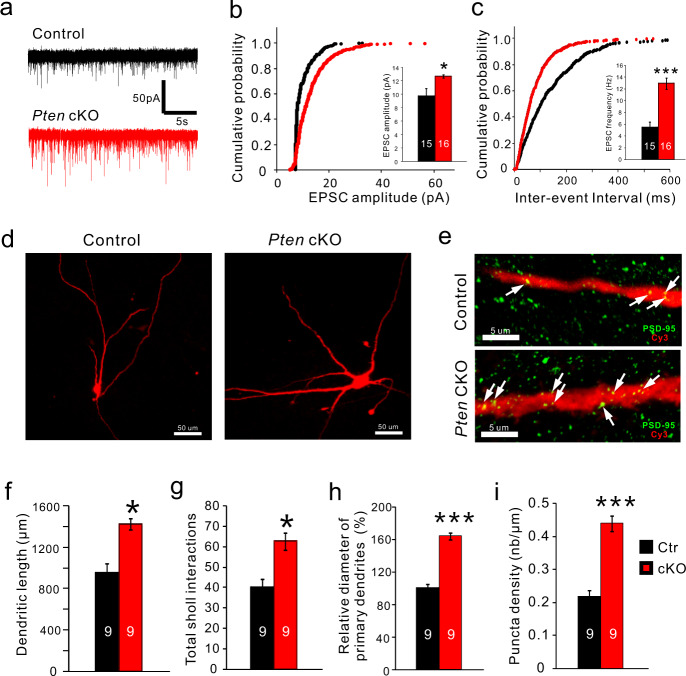
Dendritic complexity and excitatory input are increased in *Pten*-deficient 5-HT neurons. **a** Raw data traces of spontaneous excitatory postsynaptic current (sEPSC) recorded from 5-HT neurons in the dorsal raphe nucleus of control and *Pten* cKO mice. **b, c** Cumulative probability histograms show the distribution of amplitude and inter-event interval of sEPSCs events recorded from control and *Pten*-deficient 5-HT neurons. The inset shows the average amplitude and frequency of all sEPSCs are significantly increased in *Pten*-deficient 5-HT neurons (*N* = 7 mice for each group; *n* = 15 neurons in control and *n* = 16 neurons in *Pten* cKO mice). **d** Morphology of 5-HT neurons filled with biocytin (red) in control and *Pten* cKO mice. Scale bars = 50 μm. **e**. Immunostaining of PSD-95 (green) in primary dendrites of 5-HT neurons in the dorsal raphe nucleus of control and *Pten* cKO mice. Scale bars = 5 μm. **f**, **g** Total dendritic length, and branching are shown by Sholl analysis are increased in 5-HT neurons of *Pten* cKO mice compared with controls. **h** The diameter of primary dendrites of 5-HT neurons is increased in *Pten* cKO mice compared with controls. **i** The density of PSD-95^+^ puncta on primary dendrites of 5-HT neurons is increased in *Pten* cKO mice compared with controls. Nine biocytin-filled neurons from 7 mice for each genotype were used for statistical analysis in (**f, g, h**, and **i**). All the data are presented as the mean ± s.e.m. *p < 0.05, ***p < 0.001.

5-HT neurons in the DRN received prominent excitatory input from glutamatergic neurons in the prefrontal cortex (PFC)^35,36^. Both direct stimulation of glutamatergic ventromedial PFC-DRN axons and cortical deep brain stimulation induces a strengthening of excitatory synaptic input onto DRN 5-HT neurons and promotes antidepressant responses^37,38^. The decreased depression-like behaviors in *Pten* cKO mice prompted us to analyze the glutamatergic input in 5-HT neurons. To analyze the electrophysiological properties and synaptic input of 5-HT neurons in control and cKO mice, we did a whole-cell patch-clamp on YFP-expressing 5-HT neurons. *Pten*-deficient 5-HT neurons could be visualized with YFP in *Pet1-Cre;Pten*^*flox/flox*^*;Rosa-stop-YFP*. *Pet1-Cre;Rosa-stop-YFP* mice were used as control (Fig. S5a). Spontaneous excitatory postsynaptic current (sEPSC) activity was measured in the presence of bicuculline to block any inhibitory GABAergic activity (Fig. 3a). The cumulative probability plot of sEPSC amplitude demonstrated a shift toward larger sEPSC amplitude (Fig. 3b), and the average sEPSC amplitude was significantly increased in *Pten*-deficient 5-HT neurons compared with control 5-HT neurons (Fig. 3b inset). In addition, the cumulative probability plot of the inter-event interval of sEPSC demonstrated a shift toward shorter inter-event intervals, and the average inter-event interval was significantly decreased in *Pten* deficient 5-HT neurons (Fig. 3c). Furthermore, we analyzed dendritic morphology and synaptic contact on dendrites of 5-HT neurons in *Pten* cKO mice. Because YFP was only observed in the soma of 5-HT neurons in the slice, we analyzed their dendritic processes filled with 0.1% biocytin during whole-cell recording as described previously^38^. In comparison with control 5-HT neurons, *Pten-*deficient 5-HT neurons displayed an increase in total dendrite length, the diameter of primary dendrites, and dendrite branching (Fig. 3d, f–h). In addition, immunostaining of PSD-95, an excitatory postsynaptic density marker showed that the density of PSD95-labeled puncta along primary dendrites was also increased in 5-HT neurons of *Pten* cKO mice compared with that of controls (Fig. 3e, i). Thus, the complexity of the dendritic tree and excitatory synaptic input of 5-HT neurons are increased in *Pten* cKO mice.

In addition, *Pten*-deficient 5-HT neurons displayed normal resting membrane potential, increased cell capacity, and decreased input resistance (Fig. S5b–e). Action potential (AP) characteristics were determined from a single AP elicited with current injection (Fig. S5f). *Pten*-deficient 5-HT neurons showed slightly more hyperpolarized threshold, increased amplitude, and decreased AP duration, with unchanged amplitude and duration of afterhyperpolarization (Fig. S5g–k). Furthermore, α_1_-adrenergic receptor agonist phenylephrine (PE) was applied to mimic the noradrenergic input-driven firing activity of a 5-HT neuron in vivo^39^. Extracellular recordings showed that the AP firing rates were similar between control and *Pten*-deficient 5-HT neurons after bath application of 6.0 µM phenylephrine (PE), α_1_-adrenergic receptor agonist (Fig. S5l, m). Taken together, some changes such as increased cell capacity and decreased input resistance are likely to a consequence of increased soma size, and overall *Pten*-deficient 5-HT neurons remain normal electrophysiological properties and ability for AP generation.

### 5-HT innervation and axonal release is increased in *Pten* cKO mice

**Fig. 4:**
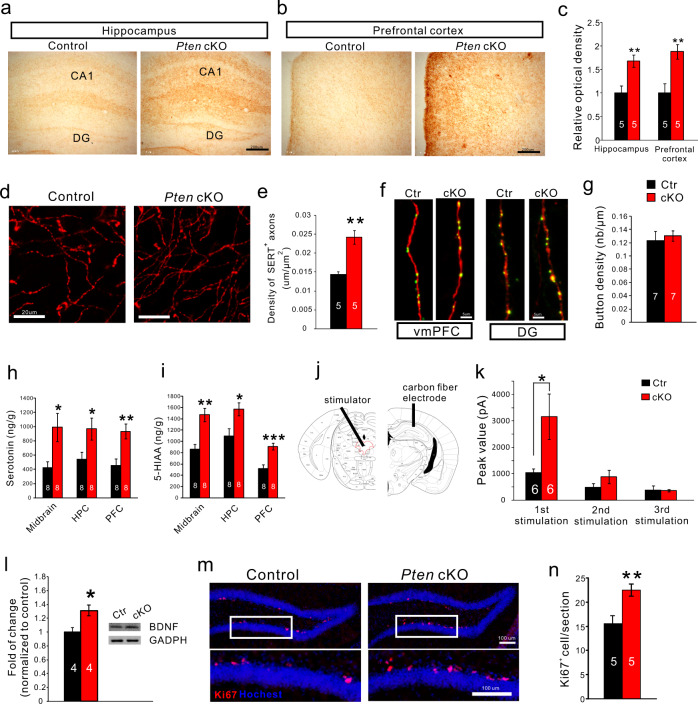
The increase of 5-HT innervation and axonal release of 5-HT in *Pten* cKO brain. **a**, **b**. SERT^+^ axons/terminals visualized with diaminobenzidine as chromogen in the hippocampus and prefrontal cortex in control and *Pten* cKO mice. Scale bars = 200 μm. **c** SERT^+^ axons/terminals shown by optical density are increased in the two regions of *Pten* cKO mice relative to control. **d**, **e** High magnification of SERT immunostaining shows individual axons/terminals in the prefrontal cortex (**d**) and comparison of the density of SERT^+^ axons/terminals there (**e**). **f** Images showing synaptophysin-fused EGFP-labeled buttons within tdTomato-labeled 5-HT axon terminals in the ventromedial prefrontal cortex (vmPFC) and dentate gyrus (DG). **g**. The density of EGFP-labeled buttons within tdTomato-labeled terminals in the ventromedial prefrontal cortex is similar between *Pten* cKO and control mice. Scale bars = 5 μm. **h**, **i** 5-HT, and its metabolite 5-HIAA are increased in the midbrain, hippocampus (HPC), and prefrontal cortex (PFC) of *Pten* cKO mice relative to control. **j**, **k** Electrical stimulation of the dorsal raphe nucleus elicits increases of 5-HT release in the hippocampus of *Pten* cKO mice compared with controls. **l** BDNF levels are increased in the hippocampus of *Pten* cKO mice. **m**, **n** The increase of Ki67^+^ cells in the subgranular zone of the dentate gyrus of *Pten* cKO mice compared with controls. Scale bars = 100 μm. Numbers of animal used are indicated. All the data are presented as the mean ± s.e.m. *p < 0.05, **p < 0.01, ***p < 0.001.

To obtain more evidence supporting the idea that the activity of *Pten*-deficient 5-HT neurons is increased, we investigated the distribution of 5-HT axon terminal in *Pten* cKO mice. 5-HT axons project throughout the brain and those to the hippocampus and mPFC are implicated in the stress-related psychological disturbances including depression^2,40,41^. Immunostaining of 5-HT transporter (SERT) showed that 5-HT axons/terminals were dramatically increased in these regions of *Pten* cKO mice (Fig. 4a–e). To analyze the presynaptic buttons of the 5-HT terminals, a Cre-dependent adeno-associated virus (AAV) expressing cytoplasmic tdTomato and presynaptic synaptophysin-fused EGFP^42^ was injected into the DRN of control and *Pten* cKO mice. The EGFP-labeled puncta along tdTomato-labeled 5-HT axon terminals were examined, and calculation of synaptophysin-fused EGFP puncta density showed no differences between control and *Pten* cKO mice (Fig. 4f, g). These results suggest that increased 5-HT terminals have a normal distribution of synaptic sites in the brain of *Pten* cKO mice.

Next, we measured 5-HT and its metabolite 5-HIAA and found that both were significantly increased in the prefrontal cortex (PFC), hippocampus, and midbrain (Fig. 4h, i) with no change in levels of norepinephrine and dopamine in *Pten* cKO mice (Fig. S3i, j). Thus, the 5-HT level is increased in the brain of *Pten* cKO mice.

We moved on to examine if the elevated 5-HT level is associated with an increase of 5-HT release in axonal terminals. Electrical stimulation (1.0 mA, 20 Hz, 10 pulses) of 5-HT neurons was performed in the DRN, and amperometric measurement of 5-HT release was done by using a carbon-fiber electrode implanted in the hippocampus of anesthetized mice (Fig. 4j). Three-successive stimulation with an interval of 5 min was applied, and a significant increase of 5-HT release was observed after the first stimulation in *Pten* cKO mice compared with controls (Fig. 4k). Thus, electrically-evoked 5-HT release in axonal terminals is elevated, further supporting the idea that 5-HT neuron activity is enhanced in *Pten* cKO mice.

Current antidepressants increase BDNF expression in brain regions strongly implicated in depression, such as the hippocampus, and the actions of antidepressants are blocked in BDNF-mutant mice^43–47^. In addition, chronic administration of the antidepressants enhances adult hippocampal neurogenesis in rodents^1,48,49^, The increased 5-HT neuron activity and reduced depression-like behaviors in *Pten* cKO mice resembles the effect of SSRI antidepressants, and we investigated the BDNF expression and neurogenesis in the hippocampus. It showed that BDNF levels were increased in the hippocampus of *Pten* cKO mice (Fig. 4l) and Ki67^+^ neural stem cells in the subgranular zone of the dentate gyrus were significantly increased in *Pten* cKO mice relative to controls (Fig. 4m, n). These results showed that *Pten* cKO mice display the increase of BDNF signaling and adult neurogenesis in the hippocampus, the known actions of the SSRI antidepressants.

### Inhibition of PTEN with bpV (pic) produces significant antidepressant effects

**Fig. 5:**
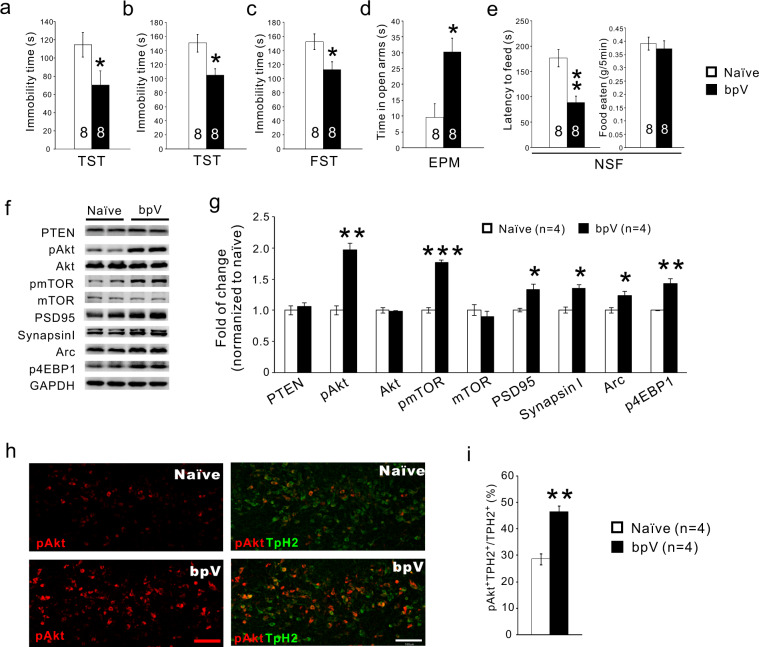
Inhibition of PTEN with bpV has significant antidepressant effects. **a** Immobility time in tail suspension test (TST) is reduced 3 days after daily administration of bpV. **b, c** Immobility time in TST and force swimming test is reduced 3 weeks after daily administration of bpV. **d**, **e**. Time spent in the open arm is increased in the elevated plus-maze test (**d**) and latency to eating food is reduced in the novelty suppressed feeding test (**e**) 3 weeks after daily administration of bpV. **f, g** Three-week systemic administration of bpV upregulates pAkt, pmTOR1, PSD-95, synapsinI, Arc, and p4EBP1 levels with no changes in PTEN, Akt, or mTOR1 in midbrain tissue containing the dorsal raphe nucleus. **h**, **i** Double immunostaining of TPH2 and pAkt shows the elevation of pAkt in 5-HT neurons in the DRN of bpV-treated mice. All the data are presented as the mean ± s.e.m. *p < 0.05, **p < 0.01, ***p < 0.001. Numbers of animal used are indicated.

Reduced depression-like behaviors and increased 5-HT neuron activity in *Pten* cKO mice suggest that reducing PTEN activity in the brain may be a potential target in treating depression. To test this idea, the PTEN inhibitor bpV (2mg/kg/day) was applied intraperitoneally to mice to inhibit PTEN^50^. The mice in the bpV group displayed significantly decreased immobility time in the tail suspension test as early as 3 days post the treatment compared with a control group (Fig. 5a), and this result encouraged us to examine the long-term effect. We found that immobility time in both the forced swimming test and tail suspension test was significantly reduced in mice treated for 3 weeks with a daily injection of the bpV (Fig. 5b, c), indicating antidepressant effects of bpV. In addition, the mice in the bpV group displayed a longer time in the open arm in the elevated plus-maze test, and a shorter latency to eat in the novelty suppressed feeding test with no changes in food consumption in the home cage (Fig. 5d, e). Spontaneous locomotor examined in the open field also showed no change. Western blotting results demonstrated activation of Akt/mTOR signaling in midbrain tissues of bpV-treated mice, including an increased level of phosphorylated Akt (pAkt), phosphorylated mTOR (pmTOR), and phosphorylated eukaryotic initiation factor 4E binding protein 1 (p4E-BP1)^51^ with unchanged total Akt and mTOR (Fig. 5f, g). In addition, bpV application also increased levels of the synaptic proteins Arc, PSD95, GluR1, and synapsin I in the midbrain tissues (Fig. 5f, g). At the cellular level, the number of pAkt^+^ 5-HT neurons was also increased in the DRN of bpV-treated mice compared with that of controls (Fig. 5h, i), showing that intraperitoneal injection of bpV is able to inhibit PTEN signaling in 5-HT neurons in the brain. Taken together, the systemic administration of PTEN inhibitor produces fast and sustained antidepressant effects in mice.

## Discussion

**Fig. 6:**
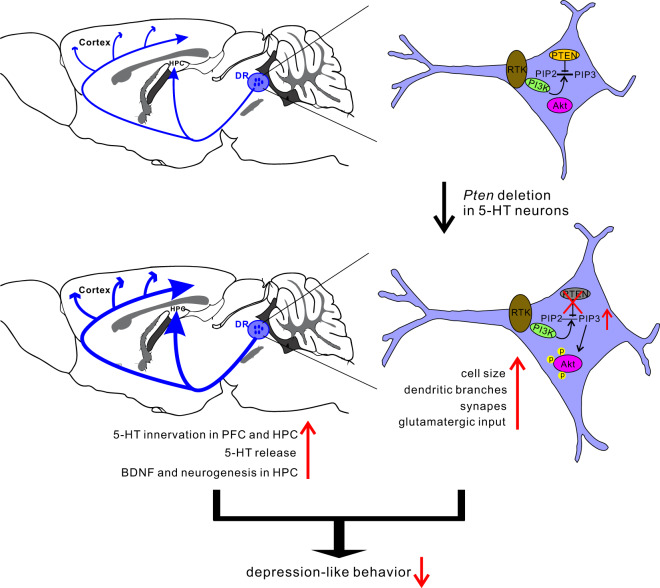
Schematic diagram demonstrating the effect of *Pten* deletion in 5-HT neurons. Environmental factors such as chronic stress upregulate PTEN expression in central 5-HT neurons and reduce its functions leading to the onset of depression. Conversely, deletion of *Pten* gene or reducing its expression in the 5-HT neurons may restore its functional and structural plasticity and display antidepressant effects, possibly via enhancing adult neurogenesis and BDNF levels in the hippocampus.

In this study, we describe a new way to increase the activity of central 5-HT neurons and demonstrate associated antidepressant effects for such manipulation. The major findings are: (i) PTEN level is elevated in central 5-HT neurons in response to chronic stress. (ii) Selective inactivation of *Pten* in 5-HT neurons increases 5-HT content and its release from axon terminals, and increased dendritic complexity of 5-HT neurons with enhanced glutamatergic input (Fig. 6). (iii) Inactivation of *Pten* leads to alterations of gene expressions underlying the morphological and functional changes of 5-HT neurons. (iv) *Pten* cKO mice and wild-type mice treated with systemic administration of PTEN inhibitor show reduced depression-like behaviors. These results demonstrate that PTEN is a critical gene in regulating the activity of central 5-HT neurons and that manipulating its expression holds an antidepressant potential.

SSRIs and other monoamine-based antidepressants remain the first line of therapy for depression. These antidepressants are designed to increase the action time of monoamines at the synaptic site thus enhancing the activity of monoamine systems in the brain. In line with that, we now report that increasing the activity of central 5-HT neurons by manipulation of its intrinsic genes is able to produce antidepressant effects, as demonstrated with the *Pten* cKO mice or with the behavioral effects of the PTEN inhibitor bpV. *Pten*-deficient 5-HT neurons display increased dendritic complexity with more excitatory synaptic contacts and increased axonal terminals with normal releasing sites. These are associated with functional alterations of intrinsic electrical properties together with increased glutamatergic input, which enables *Pten*-deficient 5-HT neurons to release more 5-HT from their axonal terminals. In addition, the lowered freezing responses in the contextual fear memory test indicated that *Pten* cKO mice are resilient to stressful stimuli, and may thus be protected from the contribution of this risk factor to the development of depression. These results suggest that reducing PTEN activity in central 5-HT neurons potentially has antidepressant effects via increasing central 5-HT neuron activity. In support of this, the increases of BDNF levels and hippocampal neurogenesis are observed in *Pten* cKO mice, and these two events are also present in chronic SSRIs-treated animals.

PTEN negatively regulates the activation of the Akt/mTOR pathway, and PTEN/Akt/mTOR signaling is implicated in the induction of synaptogenesis. Increasing evidence indicates that ketamine is a new therapeutic agent for depression. Its activity was found to be associated with fast induction of synaptogenesis and reversal of neuronal atrophy caused by chronic stress in rodents^1,2,52,53^. It should be noted that these effects are mediated by activation of Akt/mTOR pathway^2,51,54^. Furthermore, recovery of BDNF signaling, which is suppressed by chronic stress and the associated depression, is required for the antidepressant effects induced by atypical antidepressants (i.e., ketamine) and typical antidepressants (e.g., SSRIs)^1,2,55^. As part of that effect, inhibiting glycogen synthase kinase 3 (GSK3) by activation of Akt is crucial^55–57^. In addition, an increase in GSK3β activity and decreased activity of Akt have been reported in depressed but not in non-depressed suicide subjects^58^, and increased PTEN level and decreased Akt activity have been reported in the cerebral cortex and hippocampus of depressed suicide victims^13,14^. In this study, we found that chronic stress induces elevation of PTEN in 5-HT neurons, and that systemic administration of PTEN inhibitor produces antidepressant effects. Elaborating on the abovementioned findings, we propose that the PTEN-Akt-mTOR pathway is an intracellular transducer in response to chronic stress. When PTEN is over-produced for example in response to chronic stress, Akt/mTOR activity is inhibited, which in turn leads to maladaptive neuroplasticity particularly in depression-associated neuronal circuits including 5-HT neuron-related circuits, thus contributing to the development of depressive symptoms. Conversely, reducing PTEN or increasing Akt/mTOR activity is able to reverse this process and by this to lead to antidepressant effects, in agreement with the “neuroplastic hypothesis” of depression^2,6^.

High recurrence rates are one of the limitations of current monoamine-based antidepressants, which are designed to suppress the degradation or reuptake processes of monoamines. Long-term use of the antidepressants may lead to structural plasticity in the brain contributing to relieving the symptoms of depression, but it may not occur or does not maintain in some patients leading to the recurrence. Our data showed regulating *Pten* in central 5-HT neurons results in enhancements of 5-HT content and its axonal release accompanied by increased dendritic branching and excitatory input. It is likely PTEN signaling is a promising target for developing new antidepressants to reduce the recurrence rate.

## Supplementary information

Supp figures
