# Supplementary material for: Pten is a key intrinsic factor regulating raphe 5-HT neuronal plasticity and depressive behaviors in mice
Source: Transl Psychiatry. 2021 Mar 26;11:186. doi: 10.1038/s41398-021-01303-z (PMC7998026; doi:10.1038/s41398-021-01303-z)
Supplement: Supplementary file 1 — Supp figures [file 41398_2021_1303_MOESM1_ESM.pdf]

***Pten* is a key intrinsic factor regulating raphe 5-HT neuronal plasticity and depressive behaviors in mice**

Ling Chen[1,2], Wan-Kun Gong[2], Cui-ping Yang[2], Chan-Chan Shao[2], Ning-Ning Song[3], Jia-Yin Chen[2], Li-Qiang Zhou[4], Kun-Shan Zhang[4], Siguang Li[4], Zhili Huang[3], Gal Richter-Levin[5], Lin Xu[6]*, Yu-Qiang Ding[2,3,7]*

Supplementary figures

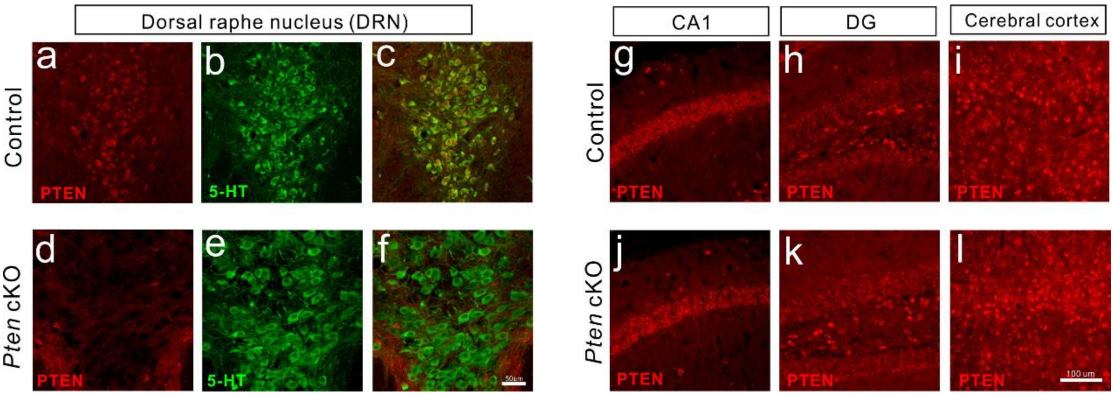

Supplementary Fig. 1. Selective deletion of PTEN in neurons expressing 5-HT.
a-c. Co-localization of 5-HT and PTEN in the dorsal raphe nucleus of control mice.
d-f. PTEN signal is absent in 5-HT^{+} neurons of *Pten* cKO mice.
g-l. Similar PTEN expression is present in the hippocampal CA1 region, dentate gyrus (DG) and cerebral cortex of control (**g-i**) and *Pten* cKO mice (**j-l**). Scale bars = 100 μm.

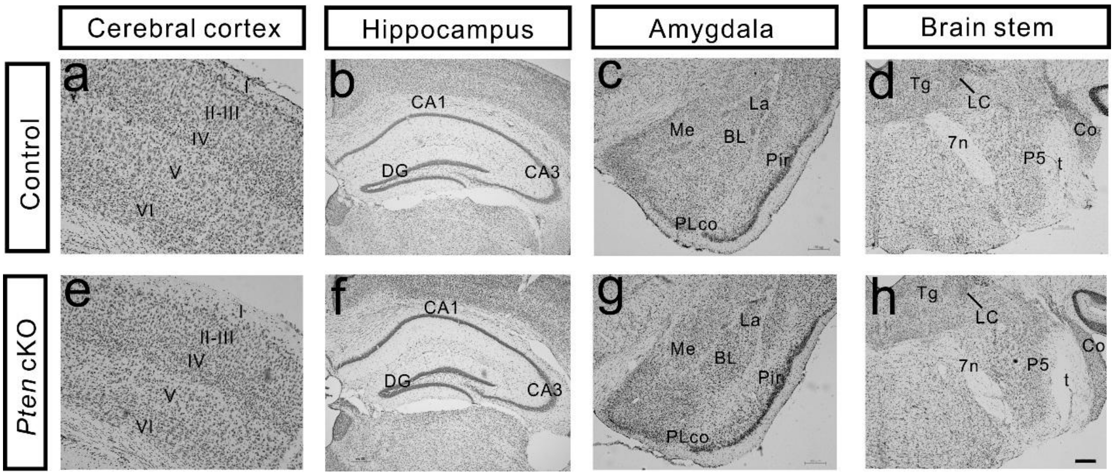

Supplementary Fig. 2. The cellular architecture shown by Nissl staining is normal in the brain of *Pten* cKO mice relative to controls.
7n, facial nerve; BL, basolateral nucleus of amygdala; CA1 and CA3, CA1 and CA3

subfields of hippocampus; Co, cochlear nucleus; DG, dentate gyrus; I-VI, cortical layers; Me, medial nucleus of amygdala; La, lateral nucleus of amygdala; LC, locus ceruleus; P5, principal nucleus of trigeminal nerve; Pir, piriform cortex; PLCo, posterolateral cortical amygdaloid nucleus; t, trigeminal tract; Tg, pontine tegmental nucleus. Scale bar = 100 μm.

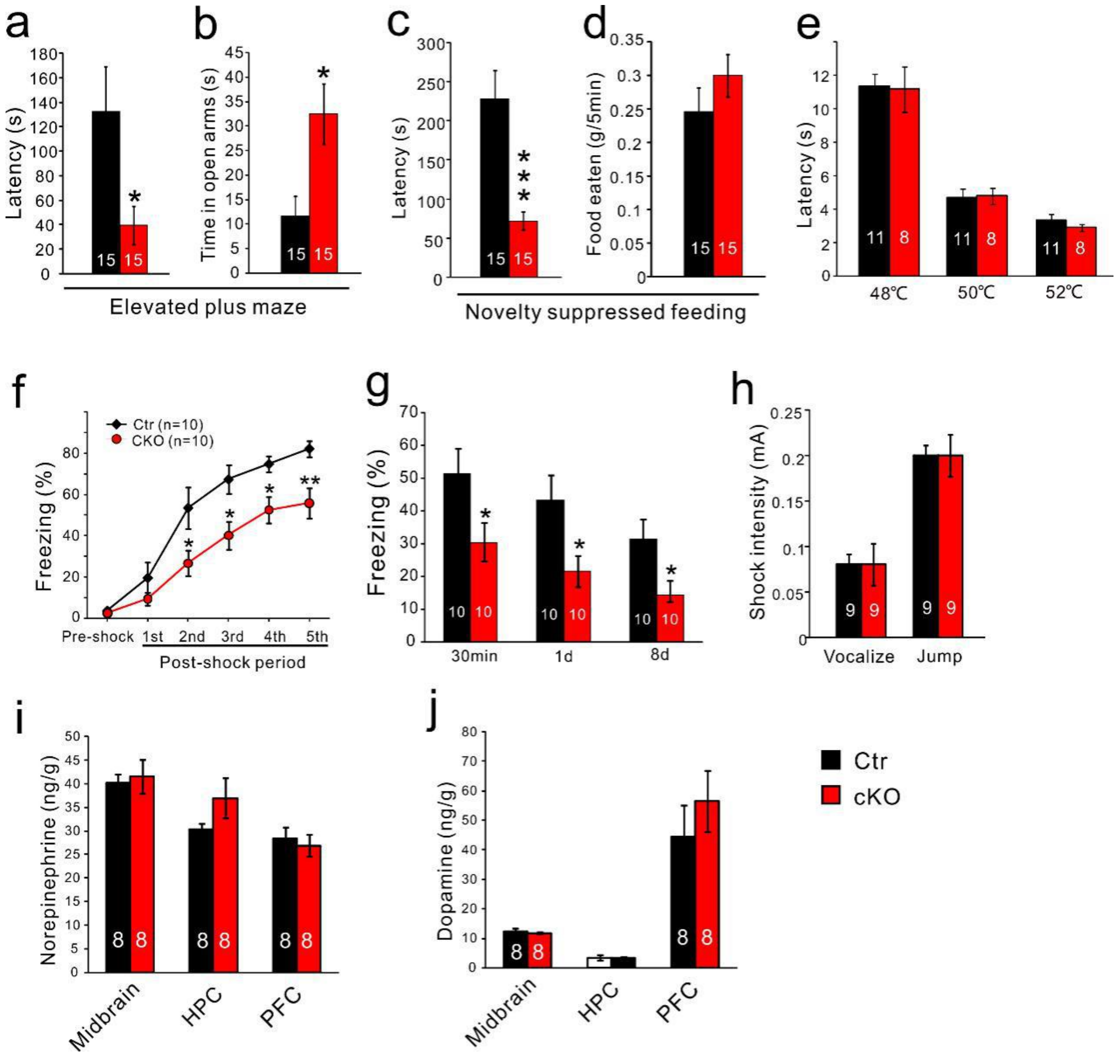

Supplementary Fig. 3. Normal somatosensory sensation and levels of norepinephrine and dopamine in *Pten* cKO mice.

a, b. The latency of entering into the open arm is reduced while time spent in the open arm is increased in *Pten* cKO mice compared with controls.

c, d. The latency of beginning eating food is reduced in the novelty suppressed feeding test in *Pten* cKO mice with no changes in food consumption compared with controls.

e, Thermal sensation shown by the withdraw latency after immersion of tail into water at indicated temperatures are comparable between control and *Pten* cKO mice.

f, g. Freezing responses are reduced in the foot shock conditioning (**f**) and retrieval of fear memory at 30 min, 1 day and 8 days post conditioning in *Pten* cKO mice compared with controls (**g**).

h, Sensitivity to foot shock revealed by increasing electric current until the occurrence of vocalize and jump in contextual fear memory test.

i, j. The levels of norepinephrine and dopamine in control and cKO brains are comparable. All the data are presented as the mean ± s.e.m. *$p<0.05$, **$p<0.01$, ***$p<0.001$. Numbers of animal used are indicated.

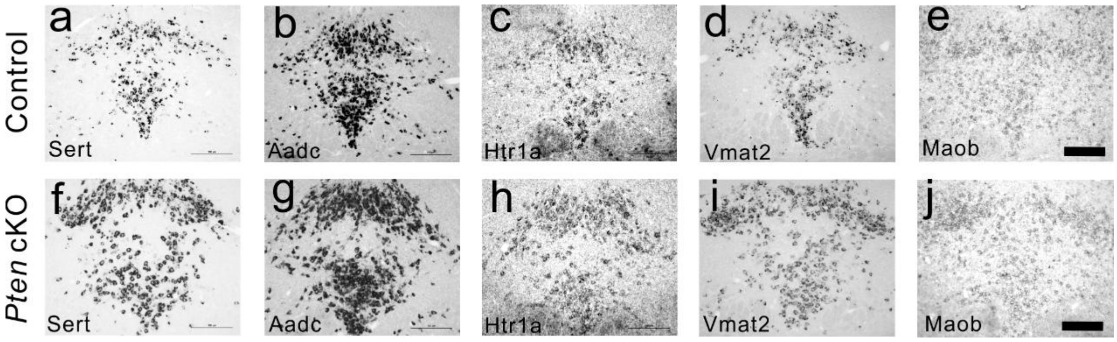

Supplementary Fig. 4. In situ hybridization shows the distribution of *Sert*, *Aadc*, *Htr1a*, *Vmat2*, and *Maob* in the DRN of control and *Pten* cKO mice.

a-e, *In situ* hybridization of *Sert*, *Aadc*, *Htr1a*, *Vmat2*, *Maob* in the DRN of control.

f-j, In situ hybridization of *Sert*, *Aadc*, *Htr1a*, *Vmat2*, *Maob* in the DRN of *Pten* cKO mice. Scale bars = 100 μm.

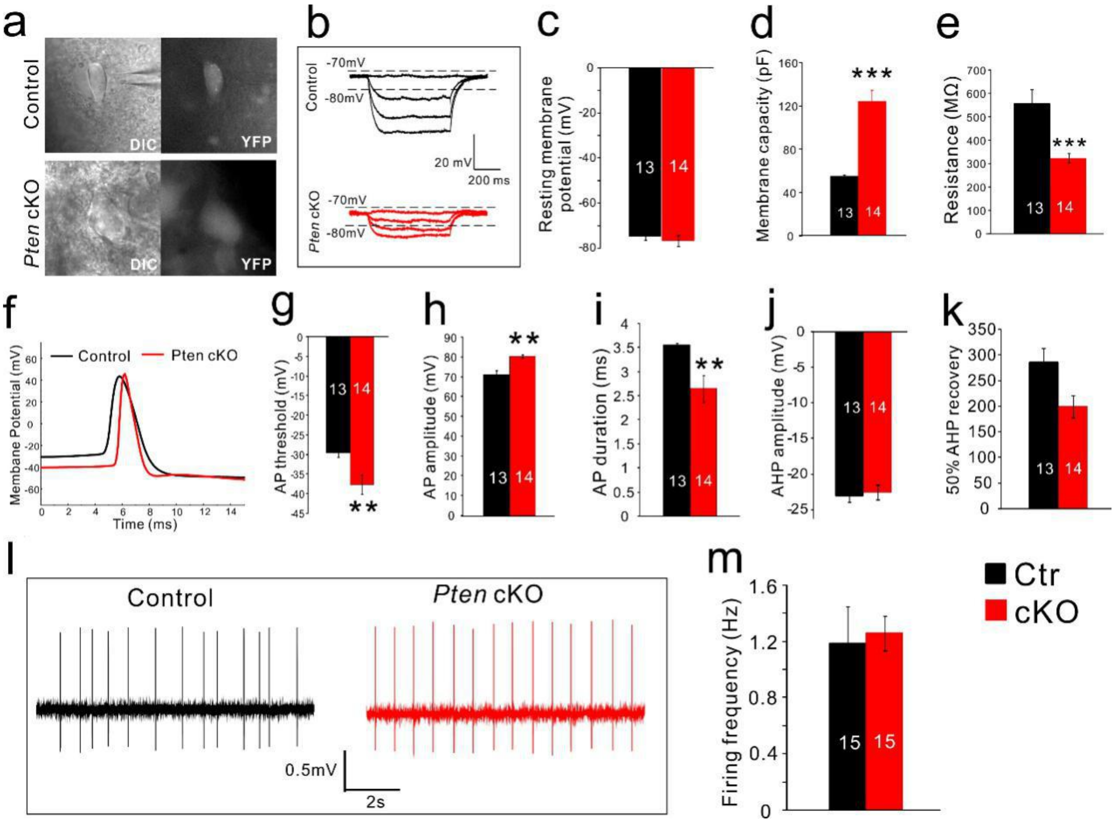

Supplementary Fig. 5. Normal resting membrane potential and the ability for action potential generation in central 5-HT neurons of *Pten* cKO mice.

a. Images show the representative YFP-labeled 5-HT neurons in the dorsal raphe nucleus of control and *Pten* cKO mice.

b. Raw data traces depicting changes in membrane potential of 5-HT neurons induced by hyperpolarizing current injection.

c. The resting membrane potential is unchanged in *Pten*-deficient 5-HT neurons.

d. The capacity of 5-HT neurons is larger in *Pten*-deficient 5-HT neurons.

e. The input resistance is decreased in *Pten*-deficient 5-HT neurons.

f. Representative recording traces showing individual action potentials (APs) induced by current injection in 5-HT neurons of control and *Pten* cKO mice.

g-i. *Pten*-deficient 5-HT neurons have more hyperpolarized threshold, increased amplitude and decreased duration of APs.

j, k. The amplitude and duration of afterhyperpolarization is unchanged in *Pten*-deficient 5-HT neurons.

l, m. AP firing rates are similar between control and *Pten*-deficient 5-HT neurons after bath application of 6.0 μM PE, α_1-adrenergic receptor agonist.

All the data are presented as the mean ± s.e.m. **p<0.01 ***p<0.001. Numbers of neurons are indicated and 3-5 mice were used in each group.
